# Controlling incisor torque with completely customized lingual appliances

**DOI:** 10.1007/s00056-020-00231-9

**Published:** 2020-05-29

**Authors:** Ons Alouini, Michael Knösel, Moritz Blanck-Lubarsch, Hans-Joachim Helms, Dirk Wiechmann

**Affiliations:** 1 Private Practice, Montpellier, France; 2 Department of Orthodontics, University Medical Center (UMG), Göttingen, Germany; 3 Department of Paediatric Dentistry and Orthodontics, Faculty of Dentistry, Universidad de La Frontera (UFRO), Temuco, Chile; 4 Private Practice, Hamburg, Germany; 5 Department of Orthodontics, University of Münster, Münster, Germany; 6 Department of Medical Statistics, University Medical Center (UMG), Göttingen, Germany; 7 Department of Orthodontics, Hannover Medical School (MHH), Hannover, Germany; 8 Private Practice, Bad Essen, Germany

**Keywords:** Malocclusion Angle class II, division 2, Tooth inclination, Incisor third-order angles, Lingual orthodontic appliances, Overbite, Malokklusion Angle-Klasse II/2, Zahnneigung, Inzisivenwinkel 3. Ordnung, Linguale kieferorthopädische Apparaturen, Overbite

## Abstract

**Purpose:**

To test the null hypothesis of no significant deviation between the center of rotation (C_ROT_) and the center of resistance (C_RES_) during space closure in Angle class II division 2 subjects achieved using a completely customized lingual appliance (CCLA) in combination with class II elastics and elastic chains.

**Methods:**

This retrospective study included 29 patients (male/female 11/18; mean age 15.6 [13–27] years) with inclusion criteria of an Angle class II/2 occlusion of least of half of a cusp, maxillary dental arch spacing, completed CCLA treatment (WIN, DW Lingual Systems, Bad Essen, Germany) in one center with a standardized archwire sequence and use of class II elastics and elastic chains only. Maxillary incisor root inclination was assessed by X‑ray superimpositions of the maxilla at the beginning (T1) and the end (T3) of CCLA treatment. Using Keynote software (Apple®, Cupertino, CA, USA), the incisor’s C_ROT_ was assessed with the point of intersection of the incisor axes (T1; T3) following vertical correction of overbite changes. C_RES_ was defined at 36% of the incisor’s apex–incisal edge distance.

**Results:**

The null hypothesis was rejected: the mean C_ROT_ − C_RES_ difference was 52.6% (*p* < 0.001). The mean C_ROT_ was located at 88.6% (min–max 51–100%) of the incisor’s apex–incisal edge distance. Although 6.9% of C_ROT_ were located between the C_RES_ and the alveolar crest, the vast majority (93.1%) were assessed between the alveolar crest and the incisal edge, or beyond.

**Conclusion:**

CCLAs can create upper incisor palatal root torque even in cases in which lingually oriented forces applied incisally to the center of resistance of the upper incisors counteract these intended root movements.

## Abstract (de)

**Ziel:**

Es wird die Nullhypothese getestet, dass bei der Korrektur der lückigen Angle-Klasse II/2-Malokklusion mit einer vollständig individuellen lingualen Apparatur („completely customized lingual appliance“, CCLA) keine signifikante Abweichung zwischen dem Rotationszentrum (C_ROT_) und dem Widerstandszentrum (C_RES_) der Oberkieferinzisivi während der Bisslagekorrektur und des Lückenschlusses mit Klasse-II-Gummizügen und elastischen Ketten auftritt.

**Methoden:**

Es wurden 29 Patienten (m:w = 11:18, mittleres Alter 15,6 [13–27] Jahre) in diese retrospektive Studie gemäß folgender Inklusionskriterien aufgenommen: Angle-Klasse II/2-Okklusion von mindestens einer halben Prämolarenbreite, lückiger oberer Zahnbogen, abgeschlossene CCLA-Behandlung (WIN, DW Lingual Systems, Bad Essen) an einem Standort mit standardisierter Bogensequenz sowie ausschließlicher Verwendung von Klasse-II-Gummizügen und elastischen Ketten zum Lückenschluss. Die Wurzelinklination der oberen Inzisivi wurde per Röntgenüberlagerungen der Maxilla zu Beginn (T1) und am Ende (T3) der CCLA-Behandlung bestimmt. Unter Verwendung der Software Keynote (Apple®, Cupertino/CA, USA) wurde das C_ROT_ der Inzisivi durch den Schnittpunkt der Schneidezahnachsen (T1; T3) nach vertikaler Korrektur therapeutischer Overbite-Veränderungen bestimmt. Das C_RES_ wurde per definitionem auf 36 % der Apex-Schneidekante-Distanz festgelegt.

**Ergebnisse:**

Die Nullhypothese wurde zurückgewiesen: Die mittlere Differenz zwischen C_ROT_ und C_RES_ betrug mehr als eine halbe Zahnlänge (52,6 %). Das mittlere C_ROT_ lag im Bereich der Zahnkrone bei 88,6 % (min–max 51–100 %) der Distanz von Apex zu Schneidekante. Zwar lagen 6,9 % der C_ROT_ zwischen dem C_RES_ und dem Alveolarkamm, doch die deutliche Mehrheit (93,1 %) befand sich im Bereich der Zahnkrone oder jenseits davon.

**Schlussfolgerung:**

Mit CCLA ist auch bei schwierigen Ausgangssituationen die kontrollierte Retraktion der Oberkieferfrontzähne mit adäquatem palatinalen Schneidezahn-Wurzeltorque möglich.

## Introduction

Occurrence of Angle class II division 2 (II/2) malocclusion varies between different populations [1]: While recent French, Swedish, and Turkish studies have reported lower prevalence rates, ranging from 1.8 to 5.4% [2–4], British and Croatian researchers have reported an incidence of 10% (British) or even 18% (Croatian population) [5, 6]. Orthodontic corrections are considered to be more difficult in those cases compared with Angle class II division 1 malocclusions, as incisor torque corrections are distinctively more difficult to implement, due to the increased and permanent labial resting pressure forces caused by the lips [7–9]. Achieving orthodontic treatment goals in those Angle class II/2 malocclusion subjects is even more challenging in combination with generalized dental arch spacing. Space closure in subjects with distal occlusion in combination with posteriorly inclined upper incisors requires forces that are diametrically opposed to the forces and moments needed to achieve adequate incisor axial and root inclination. While the use of elastic chains and class II elastics is common for simultaneous space closure and occlusal adjustments, it complicates incisor torque control in Angle class II/2 subjects by producing forces that reduce incisor root inclination without providing additional counteracting moments. Therefore, the orthodontic challenge in these subjects is aggravated by an enhancement of the retro-inclination of maxillary incisors, along with a worsening of deep bite commonly associated with Angle class II/2 malocclusion. A variety of approaches have been developed to overcome this dilemma. The segmented arch technique as described by Burstone [10] represents a sophisticated approach for simultaneous space closure by retraction and uprighting of incisors, by generating a distalizing force vector apically to the center of resistance leading to posterior movement of the incisors combined with a proclining inclination change. Also, closing-loop mechanics with nonsegmented labial archwires may be suitable for incisor retraction along with an adequate proclination, making use of appropriate gable bends mesially and distally of the retraction loops. However, despite these sophisticated approaches, the most common clinical approaches include space closure by either elastic chains or class I sliding mechanics, both of which are capable of achieving space closure, but have the immanent challenge of producing force vectors counteracting third-order incisor correction by palatal root torque required in Angle class II/2 treatments.

Consequently, the challenge in correcting this type of malocclusion using elastic power chains and class II elastics consists of creating an adequate moment that not only equalizes the detrimental forces, but also exceeds them in magnitude, in order to apply an adequate palatal torque to the incisors during space closure and class II bite correction. Using fixed orthodontic appliances, this counteracting moment (couple of forces) is commonly attempted with a rectangular archwire in the rectangular bracket slot. Based on a finite element analysis, Liang et al. [11] suggested that conventional lingual appliances are more prone to a loss of torque control of the maxillary incisors during retraction than labial appliances. They concluded that, compared with labial techniques, lingual appliances should be designed to increase lingual root torque [11].

## Study objective

The aim of this study was to evaluate the effectiveness of a completely customized lingual appliance (CCLA; slot dimensions: 0.018 × 0.025 inches ribbonwise; WIN, DW LingualSystems, Bad Essen, Germany) in creating a tooth movement, specifically upper incisor palatal root torque, in cases with generalized spacing and an Angle class II/2 malocclusion, using common space closure mechanics, such as power chains and intermaxillary elastics. We tested the null hypothesis of no significant deviation between the center of rotation (C_ROT_) and the center of resistance (C_RES_) following space closure in Angle class II/2 subjects achieved using a CCLA in combination with class II elastics and elastic chains.

## Subjects

### Inclusion and exclusion criteria

All consecutive patients treated with a CCLA (WIN; DW Lingual Systems; Bad Essen, Germany) and debonded in the time period from 1 March 2013 to 30 June 2016 in one orthodontic center (Prof. Dr. Wiechmann, Dr. Beyling and colleagues, Bad Essen, Germany) were screened for potential eligibility and were consecutively selected if they met the following inclusion criteria:

- Full permanent dentition, including eruption of second molars
- An Angle class II molar and canine occlusion of at least half of a cusp on at least one side at the beginning of Angle class II correction (T2)
- Upper incisor inclination U1/PP <115°
- Spacing in the upper dental arch
- Treatment completed with a completely customized lingual appliance (CCLA) in combination with class II elastics and elastic power chains

Subjects were excluded if they fulfilled one of the following exclusion criteria:

- Use of additional skeletal anchorage or fixed functional appliances for sagittal bite correction, such as Herbst appliances
- Dental aplasia
- Periodontal diseases or alveolar bone loss as evident in panoramic radiographs
- Treatment plan with extractions

### Included subjects

After application of the inclusion and exclusion criteria, a total of 29 Angle class II division 2 malocclusion subjects (male/female ratio 11/18; mean age at the beginning of treatment (T1): 15.6 [range 13–27] years) were included. None of the patients received skeletal anchorage or fixed functional appliances. No patient was excluded from this retrospective analysis for any other reasons such as missed appointments, lack of compliance, or missing records or informed consent to anonymized data release forms, which is occasionally seen in sample compositions of retrospective studies [12].

## Methods

### Lingual archwire sequence

All subjects were treated with a CCLA (WIN, DW LingualSystems, Bad Essen; Germany) and a standardized sequence of archwires: an initial NiTi with round diameter (diameter depending on extent of crowding 0.012″, 0.014″, or 0.016″), followed by a rectangular 0.016″ × 0.022″ NiTi archwire, and a 0.016″ × 0.024″ stainless steel wire with an incorporated maxillary canine to canine extra-torque bend of 13°. One patient received an additional 0.016″ × 0.024″ stainless steel wire with an extra-torque of 21°. A TMA 0.018″ × 0.018″ archwire was used for finishing in all treatments. Space closure and Angle class II correction was achieved with power chains (Morita Energy Chain, Rocky Mountain Orthodontics, Denver, CO, USA) and class II elastics following incorporation of the stainless steel archwires in both arches.

### Assessment of occlusion and spacing

**Fig. 1:**
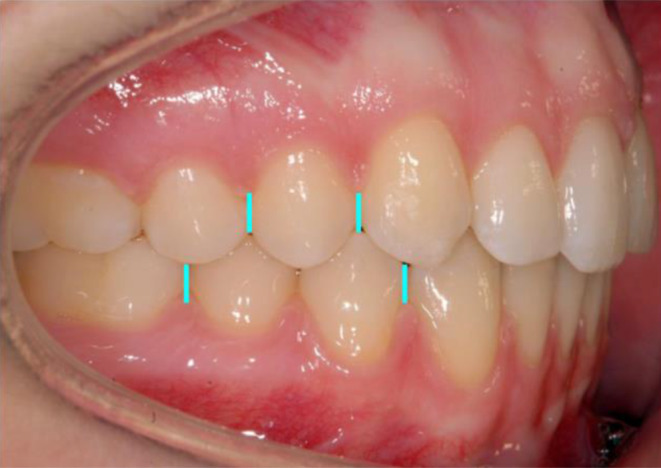
Digital, high-resolution intra-oral photographs were used to assess sagittal occlusion, with the camera axis as close as possible to 90° to the premolar/posterior tooth group. Premolars were used to assess the extent of the sagittal occlusion. See text for details Digitale hochauflösende intraorale Fotos wurden zur Bestimmung der sagittalen Okklusion verwendet, wobei die Kameraachse so nah wie möglich an der Senkrechten zur Prämolar‑/Seitenzahngruppe ausgerichtet wurde. Das Ausmaß der sagittalen Okklusion wurde anhand von Prämolaren beurteilt. Details s. Text

Sagittal occlusion was measured using high-resolution, digital, intra-oral photographs (D200, with Nikkor 105 mm; Nikon, Tokyo, Japan) at bonding (T1), following leveling and aligning and prior to using class II elastics (T2), and after debonding (T3). The photographs were taken directly, without an intra-oral mirror, using cheek retractors (Fig. 1). The camera axis was as close as possible to 90° to the premolar/posterior tooth group. We used the premolars to assess the extent of sagittal occlusion. For the assessment of the sagittal occlusion, we compared the position of the upper first premolar to that of the two lower premolars. We defined the Angle class I in our study by the upper premolar being perfectly centered with the lower premolars, equivalent to a value of 0 mm. If the cusp of the upper first premolar is centered with the mesial side of the first lower premolar, it was defined as a full Angle class II occlusion. An edge-to-edge position of the respective mesial and distal sides of upper and lower first premolar was defined as a sagittal distal occlusion of ½ cusp. The true dimensions of the assessed premolar and malocclusion on the digital photographs were assessed using a calibration technique previously employed [13]: The intraoral photographs were scaled to the corresponding plaster casts by adjusting the dimensions of the upper first premolar to its corresponding dimensions taken from direct cast measurements, using a sliding caliper. In cases in which there were different extents of sagittal occlusion on either side, the side with greater Angle class II occlusion was chosen for assessment. A neutral occlusion was defined by the first maxillary premolar centered between the lower premolars and was assigned a value of 0 mm of sagittal malocclusion. Deviations from neutral sagittal canine occlusion in the posterior direction (i.e., distal or Angle class II occlusion) were assigned positive mm values. Overbite measurements were performed on corresponding plaster cast models at T1 and at the end of treatment (T3). Wax bites taken in centric relation were utilized to correctly position upper and lower plaster casts, and the maximum overlapping was marked on the lower incisors with a pencil tip of 0.35 mm thickness. Distances between the pencil mark and the incisal edge of the incisor were taken using a sliding caliper (Dentaurum, Münchner Modell, Ispringen, Germany). Likewise, maxillary spacing was measured on the plaster casts taken at T1 and T3. All assessments of sagittal occlusion, incisor inclination, and overbite were performed manually by one operator (OA), twice, and the mean values for each of the repeated measurements were used for analysis.

### Incisor inclination assessments

#### Definition of the center of resistance

**Fig. 2:**
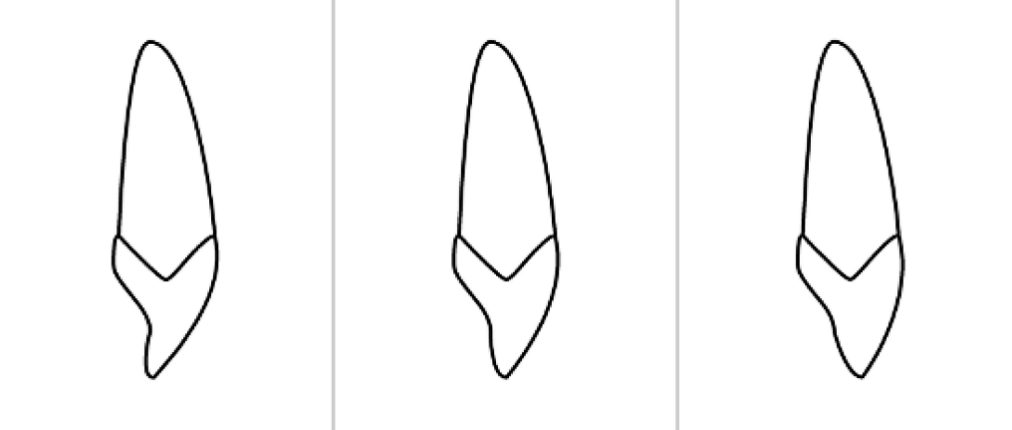
One out of three incisor templates was individually chosen in order to account for deviating crown–root angles in Angle class II/2 subjects [14, 15] Von 3 Schneidezahn-Templates wurde jeweils eins individuell ausgewählt, um der Variation der Kronen-Wurzel-Winkel bei Angle-Klasse II/2-Patienten Rechnung zu tragen [14, 15]

**Fig. 3:**
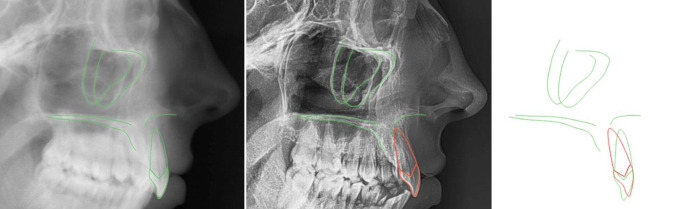
Using Cephalometrics® software, lateral X‑ray superimpositions were performed on the maxilla using the stable structures of the maxilla (i.e., the anterior part of the zygomatic processus), as suggested by Björk and Skieller [17]. Movements of the incisors were assessed with reference to the maxillary bone, as maxillary structures may be subject to alterations in position during treatment Mit Hilfe der Cephalometrics®-Software wurden laterale Fernröntgen-Überlagerungen der Maxilla angefertigt, die nach der Empfehlung von Björk und Skieller [17] die stabilen Strukturen der Maxilla (d. h. der anteriore Anteil des Processus zygomaticus) als Referenz nutzten. Schneidezahnbewegungen wurden mit Bezug auf die maxillären Hartgewebe ermittelt, da diese Strukturen während der Behandlung Positionsveränderungen erfahren können

In order to account for potential individual deviations between the root axis and the crown axis of maxillary central incisors which seen especially in Angle class II/2 subjects [14, 15], one of three digital incisor templates (with different curvatures of the cingulum) provided by Cephalometrics® software (Cephalometrics, Carpentras, France) were superimposed on and sized to these incisors using the positions of the apex and the tip (Figs. 2 and 3). The total length of every incisor measured, from apex to incisal edge, was graded in percent by a virtual ruler using Keynote software (Apple®, Cupertino, CA, USA). The template length was assigned the value of 100%.

**Fig. 4:**
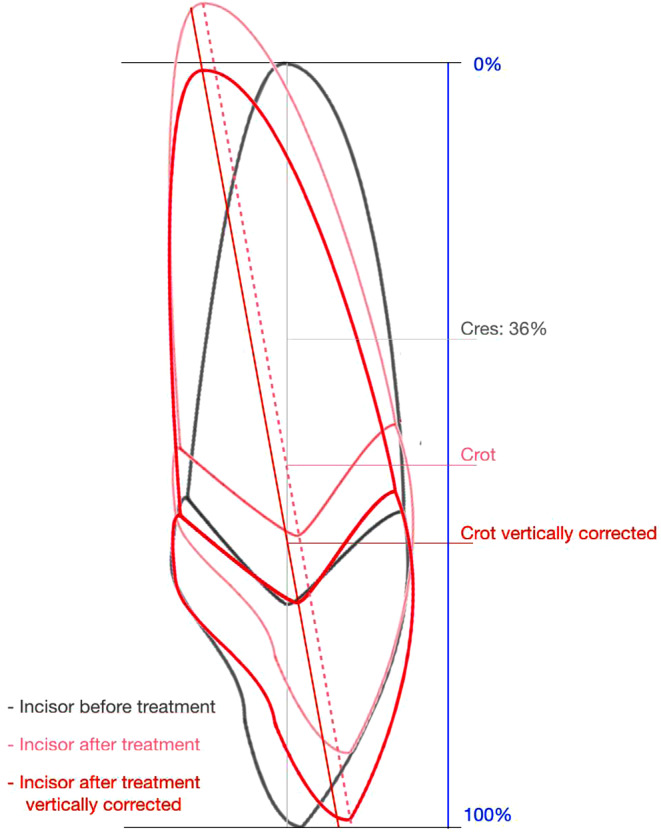
Definition of incisor landmarks Definition der Inzisiven-Referenzpunkte

Following the calculations of Burstone and Pryputniewicz who described the location of the center of resistance (C_RES_) at a point one-third of the distance from the alveolar crest to the apex [16], the C_RES_ of the tooth was defined to be located at 36% of the total length of the tooth, starting from the apex and taking account of the distance between the enamel–cement junction and the periodontally attached root (Fig. 4).

#### Primary endpoint: definition of the center of rotation

To assess the center of rotation (C_ROT_) of the incisor movement during treatment, digital pre- (T1) and posttreatment radiographs (T3) were superimposed using the stable structures of the maxilla (i.e., the anterior part of the zygomatic processus), as suggested by Björk and Skieller [17]. The C_ROT_ was assessed by the intersection of the tooth axes of the maxillary incisor before and after treatment. Using the virtual ruler, the location of the C_ROT_ was documented as a percentage of the total incisor length, starting with 0% at the apex [18]. In order to compensate for vertical proportions of incisor position changes during treatment, the posttreatment template was shifted along the incisor axis by the same amount in the opposite direction of the movement, in order to delete the vertical effect (Fig. 4). These vertically corrected C_ROT_ were separately documented and were defined as the primary endpoint of this study. For the statistical analysis, the maximum distance was set to 100%. A C_ROT_ located beyond the incisor’s edge (i.e., the transition from root torque to parallel shift) was also assigned a value of 100%. These movements always include torque control, yielding a combination of a distally oriented crown movement with an adequate counterclockwise moment produced by the bracket slot/archwire interplay.

### Method error analysis

For assessing the potential method error for assessment of the C_ROT_, measurements of 10 randomly selected patients were repeated (M1; M2) by the same examiner (OA). The mean difference of (M1 − M2) was 0.42% and no significant difference was detected (standard deviation [SD] 2.5; *p* = 0.54 by sign test). A small variation between a minimum of −2% and a maximum of 7% was considered acceptable compared to the differences seen between C_RES_ and C_ROT_.

### Statistical analysis

To assess the quality of incisor movements during CCLA treatment, the measurement data for upper incisor inclination to the palatal plane (U1/PP), sagittal occlusion, overbite, the extent of spacing of the maxillary dental arch and the location of the C_ROT_ were analyzed descriptively using mean and standard deviation (SD), as well as minimum and maximum values (min–max) at specific time points. The primary endpoint was the location of the C_ROT_ in relation to C_RES._

The potential difference between the center of rotation (C_ROT_) and the center of resistance (referred to the fixed value C_RES_ = 0.36) was analyzed using a nonparametric sign test, due to the asymmetric data distribution. The sign test was also used to assess the method error between repeated measurements of M1 and M2. The change in sagittal occlusion, U1/PP and overbite between T1 and T3 was analyzed using a paired t‑test. The significance level was set to α = 5%. All statistical analyses were derived using the statistical software SAS version 9.4 (SAS, Cary, NC, USA).

### Retrospective power calculation

With the available sample size of 29 patients and different assumed standard deviations of 20 and 30, a significance level of α = 5%, power of 90%, as well as C_RES_ = 36%, the following true expected effect sizes (∆ = expected value of C_ROT_ − C_RES_) were established: ∆ = 12.5% (assuming $SD=20$) and ∆ = 18.7% (assuming $SD=30$).

## Results

The individual occlusal features, incisor position changes, and location of C_ROT_ are provided in Table 2.

**Fig. 5:**
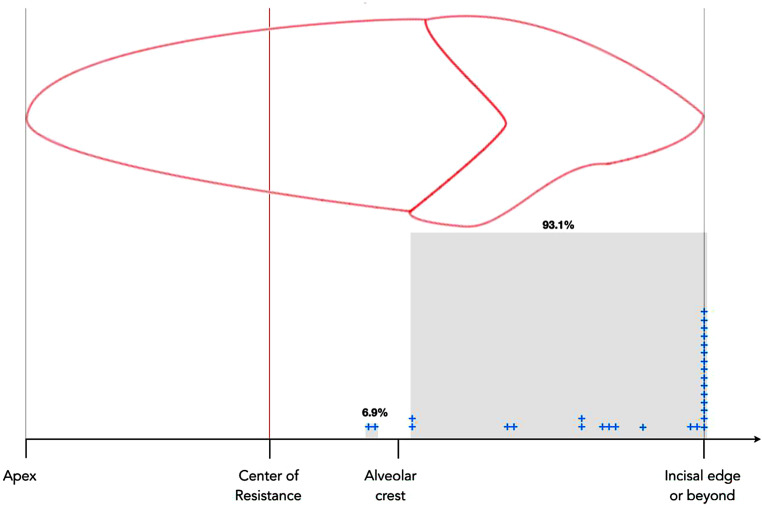
Distribution of the individual locations of the center of rotation (C_ROT_) as % of the complete incisor (0 = apex and 100 = incisor edge). No C_ROTs_ were found below or at the center of resistance (C_RES_): 0 ≥ x ≤ 36. In 2 cases (6.9%), the C_ROT_ was located between the C_RES_ and the alveolar crest (36 > x ≤ 55), while the C_ROT_ was located in 93.1% (27 patients) between the alveolar crest and the incisal edge, or beyond (55 > x ≤ 100) Verteilung der individuellen Positionen des Rotationszentrums (C_ROT_) in % der Gesamtlänge des Inzisivus, (0%=Apex, 100%=Schneidekante). Kein C_ROT_ wurde am oder apikal des Widerstandszentrums gefunden (0 ≥ x ≤ 36). In zwei Fällen (6,9 %) lag das C_ROT_ zwischen C_RES_ und dem Alveolarknochenkamm (36 > x ≤ 55), während es in 93,1 % (27 Patienten) der untersuchten Fälle zwischen dem Alveolarknochenkamm und der Inzisalkante oder darüber hinaus festgestellt wurde (55 > x ≤ 100)

The mean C_ROT_ was at 88.6% (min–max, 51–100%) of the incisor’s apex–incisal edge distance and this difference (mean C_ROT_ − C_RES_, 52.6%) was found to be statistically significant (*p* < 0.001). While 6.9% of C_ROT_ were located between the C_RES_ and the alveolar crest, the vast majority (93.1%) were assessed between the alveolar crest and the incisal edge or beyond. The distribution of the individual locations of the C_ROT_ is illustrated in Fig. 5. Table 2 also lists the result of the one sample t‑test of the null hypothesis.

### Maxillary space closure

At T1, the mean total maxillary spacing was 3.0 mm (SD 1.9; min–max 1–10 mm). At the end of CCLA treatment (T3), all maxillary spacings were closed, as planned in the target set-up (Tables 1 and 2).

**Table 1:** Patient level data for individual occlusal features, incisor position changes, and location of center of rotation (C_ROT_) with and without vertical correction by overbite changes, in % of the complete incisor (0 = apex and 100 = incisor tip) Individuelle okklusale Merkmale auf Patientenebene: Frontzahnpositionsänderungen, Lage des Rotationszentrums (C_ROT_) mit und ohne vertikale Korrektur der Overbite-Veränderungen, in Prozent der gesamten Schneidezahnlänge (0% = Apex, 100% = Schneidekante)

Patient number	Sagittal occlusion (Angle class II) mm			U1/PP °			Overbite mm			Maxillary arch spacing mm	C_ROT_ %	Primary endpoint: C_ROT_ (vertically corrected) %
	T2	T3	∆ T2–T3	T1	T3	∆ T1–T3	T1	T3	∆ T1–T3	T1		
1	3.5	0	3.5	95	114	19	4	1.5	2.5	−1.5	66	72
2	3.5	0	3.5	88.2	99.7	11.5	5	1.5	3.5	−1.5	99	98
3	6	0	6	109.5	111.3	1.8	5	1	4	−3	100	100
4	5	0	5	99.3	106.1	6.8	3.5	1	2.5	−2.5	100	100
5	3.5	0	3.5	96.5	118.8	22.3	3	1.5	1.5	−2.5	47	57
6	7	0	7	108.6	120.2	11.6	2.5	1.5	1.5	−2	52	51
7	3.5	0.5	3	108.6	108.6	0	4.5	2	2.5	−3	100	100
8	4	0.5	3.5	106.7	112.9	6.2	4.5	2	2.5	−3	88	87
9	5	0	5	94.6	112.8	18.2	4.5	1.5	3	−7	56	57
10	4	0	4	98	103.2	5.2	4	2	2	−5	100	100
11	3.5	0.5	3.5	100.2	113.3	13.1	4.5	1	3.5	−2	54	52
12	3.5	0	3.5	107.6	121.1	13.5	4.5	1	3.5	−4.5	97	100
13	5.5	0	5.5	103.5	111.6	8.1	3.5	0.5	3	−1.5	97	99
14	7	0	7	103.1	113.9	10.8	6	2	4	−3.5	90	85
15	5.5	0.5	5	104.2	108.1	3.9	5	1.5	3.5	−3	100	100
16	4.5	0.5	4	107.7	115.5	7.8	4	1.5	2.5	−2	83	82
17	3.5	0	3.5	103.5	109	5.5	3	1.5	1.5	−2	100	100
18	4.5	1.5	3	111.7	117.4	5.7	4	2	2	−3.5	87	82
19	3.5	0	3.5	110.3	111	0.7	3.5	2	1.5	−10	100	100
20	5.5	0	5.5	104.6	107.1	2.5	4	2	2	−3	100	100
21	6	0	6	105.1	107.2	2.1	5	2	3	−1.5	100	100
22	5.5	0	5.5	102.1	109.8	7.7	4	1.5	2.5	−2	100	100
23	3.5	0	3.5	97.9	99.5	1.6	6	1	5	−2	100	100
24	6.5	0	6.5	110.3	114.4	4.1	4	1	3	−2	92	86
25	3.5	0	3.5	100.3	102.8	2.5	7	1.5	5.5	2.5	100	100
26	3.5	0	3.5	114.8	115.3	0.5	4	1.5	2.5	5	100	100
27	4.5	0.5	4	95.4	104.9	9.5	5	2	3	1	67	71
28	4	0.5	3.5	103.1	118	14.9	3.5	1	2.5	1	96	91
29	3.5	0	3.5	100.8	108.2	7.4	6	2	4	3	100	100

Patient #7 showed perfectly parallel positions for the upper incisor before and after treatment, with a C_ROT_ in infinite distance (∞), and patient #19 almost perfectly parallel positions of the upper incisor axis in T1–T3 comparisons. The center of rotation of this movement is in a finite distance (near ∞), but too far to calculate a measurement value. Both incisors #7 and #19 were displaced distally, indicating adequate torque control. All values below the tip of the crown were set to 100%

**Table 2:** Descriptive statistical summary of occlusal features, incisor position changes, and location of center of rotation (C_ROT_) with and without vertical correction by overbite changes, in % of the complete incisor (0 = apex and 100 = incisor tip) Zusammenfassung der deskriptiven Statistik okklusaler Charakteristika, Positionsänderungen der Schneidezähne und Position des Rotationszentrums (C_ROT_) mit und ohne vertikale Korrektur durch Overbite-Veränderungen, in Prozent der gesamten Schneidezahnlänge (0% = Apex, 100% = Schneidekante)

*Sagittal occlusion* *(Angle class II), mm* mean ± SD [min, max]	T2	4.55 ± 1.17 [3.5, 7]
	T3	0.17 ± 0.33 [0, 1.5]
	∆ T2–T3	4.4 ± 1.23 [3, 7] *p* < 0.001^b^
*U1/PP, °* mean ± SD [min, max]	T1	103.14 ± 6.07 [88.2, 114.8]
	T3	110.89 ± 5.74 [99.5, 121.1]
	∆ T1–T3	7.74 ± 5.89 [0, 22.3] *p* < 0.001^b^
*Maxillary arch spacing, mm* mean ± SD [min, max]	T1	2.97 ± 1.89 [1, 10]
*Overbite, mm* mean ± SD [min, max]	T1	4.38 ± 1.01 [2.5, 7]
	T3	1.52 ± 0.43 [0.5, 2]
	∆ T1–T3	2.88 ± 1 [0, 5.5] *p* < 0.001^b^
*C*_*ROT*_*, %* mean ± SD [min, max]		88.65 ± 17.4 [47, 100] *p* < 0.0001
*Primary endpoint:* *C*_*ROT*_* (vertically corrected), %* mean ± SD [min, max]		88.62 ± 16.47 [51, 100] *p* < 0.001^a^

*SD* standard deviation, *min* minimum, *max* maximum

^a^Sign test

^b^Paired t‑test

### Angle class II correction

Mean sagittal malocclusion as measured on the worse side at T2 (following leveling and aligning) was 4.6 mm distal occlusion (SD 1.2; min–max 3.5–7 mm) which had improved in all patients to a mean 0.2 mm (SD 0.3; min–max 0–1.5 mm) at T3. At T3, 21 out of 29 subjects had a perfect Angle class I occlusion (0 mm), whereas 7 patients (24.1%) showed a minor distal occlusion of 0.5 mm and one subject (3.4%) had a distal occlusion of 1.5 mm. The mean sagittal correction measured on the worse side was 4.4 mm (SD 1.2; min–max 3–7 mm; Tables 1 and 2).

### Overbite correction

All of the 29 patients had a deep bite (>2 mm) at T1. Deep bite correction was achieved in all patients: at T1, the mean overbite was 4.4 mm (SD 1.0 mm), which decreased significantly (*p* < 0.001) by a mean difference of 2.9 mm (SD 1.0 mm) to a mean 1.5 mm (SD 0.4 mm; Tables 1 and 2).

### Treatment duration

Treatment duration varied from 16–43 months, with a mean value of 28 months (SD 3.5 months) between CCLA bonding and debonding.

## Discussion

### Definition of third order tooth movements

In general, there are three different ways to improve the inclination of a retroclined incisor. If a single force is applied to the crown of the tooth in an anterior direction, the crown is tipped in the buccal direction. Without the simultaneous application of a couple of force with opposite directions (a moment), the tooth will rotate with a center of rotation located inside the root. The location depends on the distance of the applied force to the center of resistance. Some of these types of tooth movements meet the definition of *uncontrolled tipping* and are typically achieved with a variety of removable appliances or with fixed appliances in combination with round archwires. At the end of such tooth movement, the inclination of the incisor is more positive and, as the crown of the tooth is tipped to the buccal side, space is created, leading to an increased arch length and an improved incisor inclination. However, it is worth noting that this improvement in incisor inclination is not equal to a (root) torque movement or a bodily movement. To achieve a torque movement, the combination of a force and a moment (couple of forces in opposite directions, e.g., a force shifted apically to the C_RES_ by a moment created by the segmented arch approach resulting in a palatal root torque) is necessary. Only this combination allows for shifting the center of rotation towards the incisal edge. To achieve movement such as a *root torque* (torque movement, bodily movement as torque) with a center of rotation close to or at the incisal tip [16], a buccally oriented force on the crown of the tooth has to be combined with a clockwise moment (buccal root torque), or a palatally oriented force on the crown of the tooth has to be combined with a counterclockwise moment (palatal root torque). Typically, buccal root torque is needed in the maxillary anterior segment for dentoalveolar compensation of an Angle class III malocclusion, whereas palatal root torque in the same segment is needed for most of the nonsurgical Angle class II corrections. If the inclination of a retroclined incisor is corrected by a palatal root torque, no space opening is observed, as the crowns do not move buccally. The third possibility to improve the inclination of a retroclined incisor is *controlled tipping* of the crown to the buccal side. In this case, the center of rotation is shifted to the apex.

Putting aside these fundamental orthodontic rules, virtually impossible results have been reported by some authors mainly during the past 7 years, regarding the alleged torqueing capabilities of some kinds of clear aligner treatment (CAT) [19–21]. A closer look at the methods applied in these studies has revealed an ambiguous use of the term “torque movements”, since they measured the inclination of the labial surface of the incisors on digital dental arches and misinterpreted each inclination change of the labial surface as a bodily movement of torque. The orthodontic community should indeed be aware of the unreflective repetition of this kind of mistake—even in systematic review articles about CAT.

### Creating a couple of forces with opposite directions

The importance of palatal root torque and/or correct incisor inclination for dental arch length and the achievement of a proper molar relationship has been the subject of many studies [22–25]. With fixed appliances, a torque moment is created by the rectangular archwire’s action to untwist when engaged in a bracket slot [26], thereby generating a *couple* of forces with opposite directions or moment that change the inclination of the incisor [25, 27]. Being a free vector, this moment is subject to spatial variability and may be located on any point along the long axis of the tooth, or outside of the tooth. Based on this knowledge, Andrews developed the straight-wire appliance using angulated, pre-adjusted bracket slots [28]. It is, however, well known that torque expression is highly dependent on archwire dimensions versus slot size [29]. Commonly used conventional labial brackets are manufactured using the technique of metal injection molding. The initially molded bracket—the so-called “brown part”—is about 30% larger than the final bracket and is made of compressed metal powder. During the sintering process, this oversized bracket is heated up very close to its melting temperature and shrinks in a very uncontrollable way, resulting in high slot tolerances in both directions: positive and negative. In order to avoid situations in which archwires do not fit into undersized bracket slots (negative tolerance), the target slot size is set to a higher value, aggravating the average oversize of the final bracket slot to values of up to 20% or more [29]. The consequences of increasing torque play by shifting the manufacture of orthodontic brackets from milling or precision casting to metal injection molding (MIM) have been discussed extensively [30–33].

### Importance of torque control and lingual treatment

In lingual orthodontic treatment, third order control is of substantial importance for achieving precision in finishing [34–37]. In a finite element analysis, Liang et al. [11] simulated and compared retraction of incisors with labial and lingual appliances and found that it was critical to control the moment/force ratio and increase lingual root torque properly with lingual appliances. It is noteworthy that, to the best of our knowledge, this topic has been previously addressed on the evidence level of in vitro experiments and retrospective studies, but not with clinical prospective study or randomized, controlled trial designs that addressed outcome differences when comparing labial, lingual or aligner technique approaches to meet a higher level of evidence. In order to address these issues of controlling the moment/force ratio adequately, the bracket slots of the CCLAs used in this study were made by high-speed milling with manufacturing tolerances of only up to ±2 μm (<0.5%) [38]. As a consequence, the magnitude of torque expression with these appliances compared to conventional labial or lingual brackets can be easily controlled by the orthodontic specialist [37, 39–45]. In order to account for torque play, the undersized maxillary rectangular 0.016″ × 0.024″ stainless steel archwires of the CCLA used here during space closure were provided with an incorporated canine-to-canine extra torque bend of 13°, and, in the case of one patient, 21°. The use of the 0.016″ × 0.024″ stainless steel archwires with built-in extra torque bends is crucial for maintaining the necessary moment to counteract and overcome the crown tipping forces in the palatal direction generated using class II elastics and power chains during space closure.

### Location of the center of resistance

According to calculations made by Burstone and Pryputniewicz, the center of resistance (C_RES_) of an upper incisor is located approximately at a point one-third of the distance from the alveolar crest to the apex [16]. Likewise, Graber et al. [34] reported that the center of resistance in a single-rooted tooth with a parabolic shape should be calculated by multiplying the distance from the alveolar crest to the apex by 0.33. Proffit et al. [43] took into consideration the condition of the periodontal tissues and described a tooth’s center of resistance to be slightly more apical, i.e., “at the approximate midpoint of the embedded portion of the root (i.e., about halfway between the root apex and the crest of the alveolar bone)”. Due to the young age of the subjects included in our study and the absence of periodontal diseases or bone loss, the alveolar crest was assigned to be located about 2 mm below the enamel/cement junction [16]. The alveolar crest was approximated by a line, and 2 mm was subtracted (Fig. 4).

### Null hypothesis

The null hypothesis of no significant deviation between the center of rotation (C_ROT_) and the center of resistance (C_RES_) during space closure in Angle class II/2 subjects was rejected. While the center of resistance of the tooth was located at 36% from the total length of the tooth starting from the apex, the mean C_ROT_ was at 88.6% (min–max, 51–100%). This difference (mean C_ROT_ − C_RES_, 52.6%) was found to be statistically significant (*p* < 0.001, Table 2).

In our study, all C_ROTs_ were found to be located above the incisor’s C_RES_ in the incisal direction, i.e., they were shifted towards the incisal edge or beyond, indicating that neither controlled nor uncontrolled incisor tipping occurred in the treated individuals.

We corrected for vertical (overbite) changes in tooth position during treatment by measuring the vertical position of the incisor before and after treatment and adjusted the center of rotation along the long axis of the tooth with the same distance. One of three incisor templates was individually chosen to account for deviating crown–root angles in Angle class II/2 subjects ([14, 15]; Fig. 2). The same template that was used for the baseline assessment of each incisor was also used for the second assessment of the respective incisor, in order to avoid within-subject changes in incisor shape and dimensions.

### Limitations

The calculation of the incisor’s C_RES_ as one factor to describe the nature of the achieved tooth movements was based on calculations made by Burstone and Pryputniewicz [16]. As the location of the C_RES_ is multifactorially influenced by tooth morphology and the properties and condition of the individual periodontum, slightly different descriptions of the C_RES_ have been provided by various authors [34, 46], and the extent to which the three-dimensional situation can be generalized has been discussed [47, 48]. However, the definition of the root centroid or C_RES_ used here is widely accepted in orthodontic literature and is considered to be a valid orientation to describing incisor movements.

## Conclusions

- Despite the use of elastic chains and class II elastics that are known to worsen incisor torque control in Angle class II/2 subjects, no uncontrolled incisor tipping occurred in the treated individuals.
- While 6.9% of center of rotation (C_ROT_) were located between the center of resistance (C_RES_) and the alveolar crest, the vast majority (93.1%) were located between the alveolar crest and the incisal edge or beyond, indicating adequate root torque control by the completely customized lingual appliances (CCLA).
- CCLAs can create upper incisor palatal root torque even in cases in which lingually oriented forces applied incisally to the center of resistance of the anterior upper teeth counteract these intended root movements.
